# Mortality of lung cancer as a second primary malignancy: A population‐based cohort study

**DOI:** 10.1002/cam4.2172

**Published:** 2019-04-16

**Authors:** Lei Deng, Hrönn Harðardottír, Huan Song, Zhengrui Xiao, Changchuan Jiang, Qian Wang, Unnur Valdimarsdóttir, Haiying Cheng, Billy W. Loo, Donghao Lu

**Affiliations:** ^1^ Department of Thoracic Oncology Cancer Center, State Key Laboratory of Biotherapy West China Hospital Sichuan University Chengdu Sichuan P.R. China; ^2^ Laboratory of Molecular Diagnosis of Cancer, Clinical Research Center for Breast West China Hospital Sichuan University Chengdu Sichuan P.R. China; ^3^ Department of Medicine Jacobi Medical Center Albert Einstein College of Medicine Bronx New York USA; ^4^ Department of Respiratory Medicine Landspitali University Hospital Reykjavik Iceland; ^5^ Faculty of Medicine Center of Public Health Sciences University of Iceland Reykjavik Iceland; ^6^ Department of Internal Medicine Icahn School of Medicine at Mount Sinai New York City New York USA; ^7^ Department of Medical Epidemiology & Biostatistics Karolinska Institutet Stockholm Sweden; ^8^ Department of Epidemiology Harvard T.H. Chan School of Public Health Boston Massachusetts USA; ^9^ Division of Medical Oncology Department of Medicine Montefiore Medical Center/Albert Einstein College of Medicine Bronx New York USA; ^10^ Department of Radiation Oncology Stanford University Stanford California USA

**Keywords:** cohort study, lung cancer, mortality, prognosis, second primary

## Abstract

Lung cancer as a second primary malignancy (lung‐2) is increasingly common, but its prognosis is poorly understood. This study aims to examine the overall and cancer‐specific survival of patients diagnosed with lung‐2 compared to lung‐1. Primary lung cancer patients diagnosed from 1988 to 2014 in the Surveillance, Epidemiology, and End Results (SEER) program were included. Lung‐2 was identified in patients with a previous diagnosis of nonlung primary malignancy in SEER. Hazard ratios (HRs) of overall and lung cancer‐specific mortality were estimated among patients with lung‐2 compared to lung‐1, adjusting for age and calendar period at diagnosis, sex, race, socioeconomic status, tumor stage, histology, tumor grade, and treatment. A total of 679 541 and 85 758 patients were identified as lung‐1 and lung‐2, respectively. Compared to lung‐1, patients with lung‐2 were more likely to be diagnosed at localized stage, with smaller primary tumor, and treated with surgery. Lung‐2 patients were at lower risk of lung cancer‐specific mortality in the first 5 years (HR, 0.77; 95% CI, 0.76‐0.78 at <1 year; HR, 0.87; 95% CI, 0.86‐0.89 from 1 to <5 years) but at higher risk thereafter (HR, 1.32; 95% CI, 1.27‐1.37 from 5 to 10 years), independent of tumor characteristics and cancer treatment. Similar pattern was found for overall mortality, although the survival benefit was restricted to the first year after diagnosis. Patients diagnosed with lung‐2 face a favorable lung cancer‐specific survival within the early period after diagnosis. A conservative approach to manage lung‐2 solely based on malignancy history is not supported.

## 1 INTRODUCTION

As the survival of cancer patients improves, second primary malignancy has been increasingly diagnosed among cancer survivors.^1^ Many cancer survivors are at increased risk of developing second primary lung cancer (lung‐2), due to shared hazardous lifestyles, cancer treatment, and intrinsic genetic factors of the first primary ones.^2^, ^3^ However, it is largely unknown whether lung‐2 itself has a different aggressiveness compared to lung cancer as the first primary malignancy (lung‐1).

Clinical decision‐making for patients presenting with lung‐2 sometimes can be challenging because of the limited and conflicting information on prognosis. A study of Hodgkin lymphoma patients who developed lung‐2 showed inferior overall survival of lung‐2 compared to lung‐1,^4^ whereas the overall survival of lung‐2 among breast cancer survivors was not worse compared to lung‐1.^5^ The reduced overall survival (if any) is, however, not surprising, given the non‐negligible mortality contribution of the first primary cancer, and does not necessarily indicate more aggressive behavior of lung‐2.

To better capture the prognosis of lung‐2, lung cancer‐specific mortality, rather than overall survival, is indeed the optimal measure. Yet, such evidence on lung‐2 is currently lacking. We therefore utilized the well‐established Surveillance, Epidemiology, and End Results (SEER) database to examine the risks of lung cancer‐specific and overall mortality among patients with lung‐2 compared to lung‐1, and whether tumor, clinical, and demographic factors could potentially modify such risks.

## 2 MATERIALS AND METHODS

### 2.1 Study population

Based on National Cancer Institute's SEER program, we conducted a population‐based cohort study of patients with primary lung cancer diagnosed between January 1, 1988 and December 31, 2014 in US. SEER has collected information on demographic, tumor and clinical characteristics, and follow‐up from nine registries since 1997 (covering about 9.4% of the US population) and expanded to 13 registries in 1992 (about 13.4% of the US population). We identified 989 150 patients with primary lung cancer confirmed by pathological diagnosis. The following patients were further excluded: diagnosed from autopsy (N = 20 602), younger than 18‐year‐old (N = 188), with no birth year (N = 21), and without accurate follow‐up (N = 101 814).

### 2.2 Ascertainment of first and second primary lung cancer

SEER has stringent criteria to define multiple primary neoplasms. Because pathological diagnosis is required for inclusion, it is less likely that lung‐2 identified in SEER constitute metastases from the first primary malignancy unless the first primary is lung cancer. Per SEER rules, an uncertain case is considered as a single primary unless proven as a new primary.^6^

Among the remaining 866 525 patients, 186 984 were recorded as not their first malignancy. By linking to the previous malignancy diagnoses in SEER, we further excluded patients with lung cancer as the third or above primary malignancy (N = 18 152), and patients with no information on previous malignancy diagnosis (N = 63 484). Because of the uncertainty of separating a lung‐2 from intrapulmonary metastases of lung‐1 and the difficulty to determine death from lung‐1 or lung‐2 patients with a prior history of lung cancer were also excluded (N = 19 590). Thus, lung‐2 in this analysis represents primary lung cancer as a second malignancy after a prior nonlung malignancy. Finally, we identified 765 299 patients with primary lung cancer, including 85 758 lung‐2 and 679 541 lung‐1.

### 2.3 Ascertainment of mortality

We studied lung cancer‐specific mortality as primary outcome and overall mortality as secondary outcome. All patients were followed from cancer diagnosis until December 31, 2014 or death, whichever came first, by linking registries through health care institutions or by directly contacting patients. The cause of death is derived from death certificate with algorithms to identify a single, disease‐specific cause. The algorithm considers tumor sequence, site of the cancer diagnosis, and comorbidities.^7^

### 2.4 Statistical analysis

First, we described the demographic, tumor, and clinical characteristics between patients with lung‐1 and lung‐2. The association of lung‐2 with tumor and clinical characteristics was analyzed by using binary or multinomial logistic regression. Demographic characteristics were adjusted for in the analysis of tumor characteristics, while tumor features were additionally controlled for in the analysis of clinical characteristics. Education level and cost of living were obtained at county level with data annexed to SEER.

We plotted the cumulative mortality rates by causes of death among patients with lung‐1 and lung‐2 from diagnosis to 10 years afterwards. Because the proportional hazards assumption was violated, we derived the hazard ratios (HRs) and 95% confidence intervals (CIs) of overall and lung cancer‐specific mortality among lung‐2 from a flexible parametric survival model,^8^ which allows HRs to change over time. To address the concern of misclassification of causes of death (eg, deaths due to lung‐2 attributed to the first primary malignancy or vice versa), we additionally analyzed the association with any cancer‐specific mortality.

In the aforementioned analyses, we always adjusted for demographic characteristics in a first model (model A) and additionally controlled for tumor and clinical characteristics—as potential mediators (eg, patients with lung‐2 are more likely to be diagnosed at earlier stage and subsequently have better survival)—in a second model (model B). Age was used as a continuous variable, while other factors were categorized. Our emphasis was though on reporting associations independent of tumor and clinical characteristics, thus we only applied model B on the analyses below.

Given the heterogeneous HRs over time, the subsequent analyses on factors of first malignancy and lung cancer were separately performed within different follow‐up periods (0 to <1 year, 1 to <5 years, and 5 to 10 years after diagnosis), using Cox proportional hazards models. To address the concern of higher possibility of dying from first primary malignancy among patients with lung‐2, we estimated the HR of lung cancer‐specific mortality using a competing risk model.^9^

We performed all analyses in STATA 14.2 (StataCorp LP). *P* < 0.05 indicated statistical significance. The study was approved by the Biomedical Research Ethics Committee at West China Hospital.

## 3 RESULTS

### 3.1 Patients and clinical characteristics

A total of 679 541 and 85 758 patients had lung‐1 and lung‐2, respectively, with a median follow‐up of 0.6 year (0‐26.9 years). Lung‐2 accounted for 8.8% of the total new cases in 1988‐1992 and 14.5% in 2011‐2014 (Figure S1). Compared to lung‐1, patients with lung‐2 were older and diagnosed more recently (Table S1), more likely to be diagnosed at localized stage, with smaller tumor size, squamous, and well‐and‐moderately differentiated tumors (Table S2). Lung‐2 patients were more likely to undergo surgery, but less likely to receive radiation therapy (Table S2). Among lung‐2 patients, the median time from the first malignancy to lung‐2 diagnosis was 4.8 years. The most common three sites of first malignancy were prostate, breast, and colon and rectum (Figure S2).

### 3.2 Mortality risk of lung‐2 relative to lung‐1

**Figure 1:**
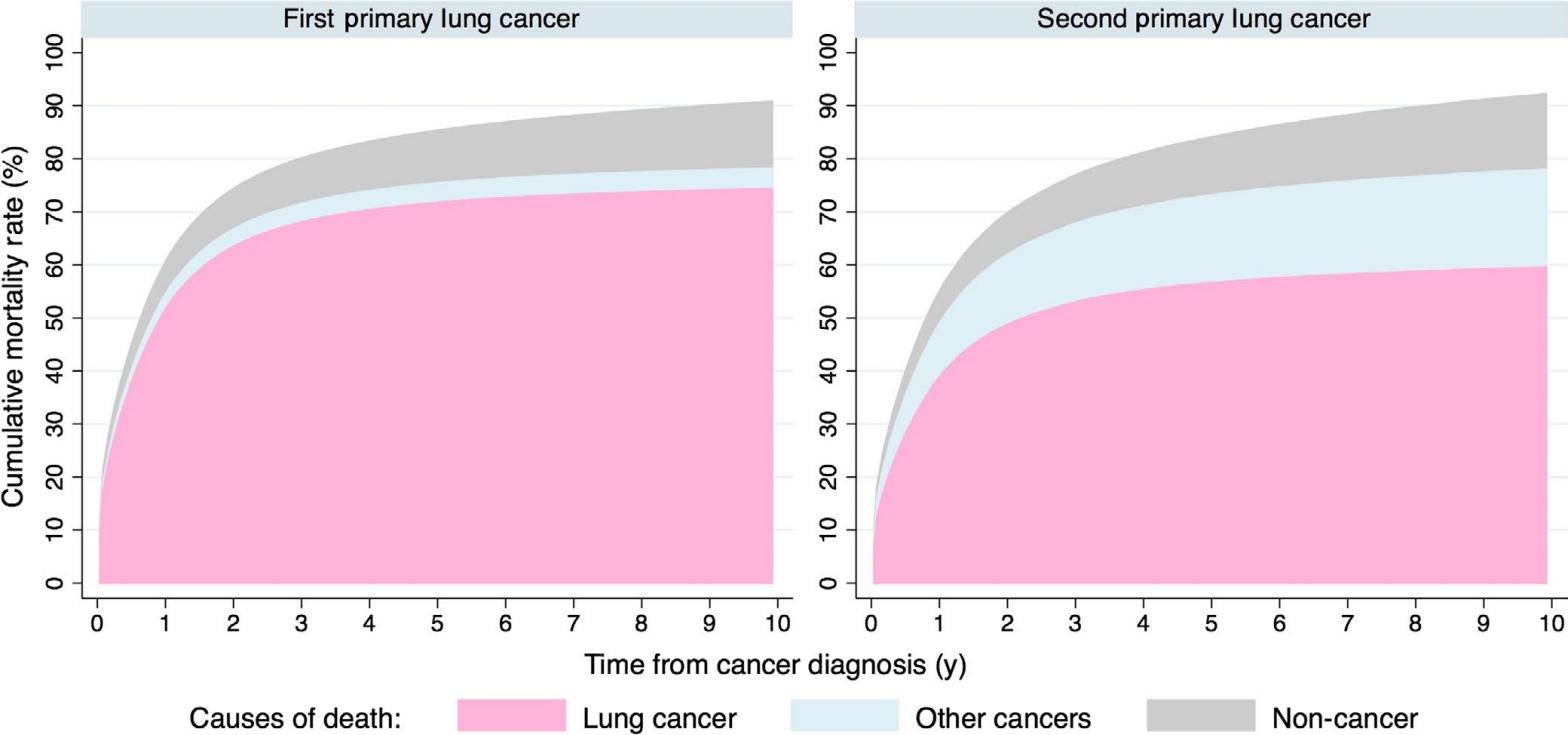
Cumulative mortality rates by causes of death among patients with first and second primary lung cancers from cancer diagnosis to 10 years afterwards: a population‐based cohort study in US, 1988‐2014

A total of 551 134 (81.1%) and 69 714 (81.3%) lung‐1 and lung‐2 patients died during follow‐up. Compared to lung‐1, patients with lung‐2 had lower cumulative mortality rate due to lung cancer, but higher cumulative mortality rate due to other cancers, from diagnosis to 10 years afterwards (Figure 1). The cumulative mortality rate of all causes was lower in lung‐2 until almost 7 years after diagnosis.

**Figure 2:**
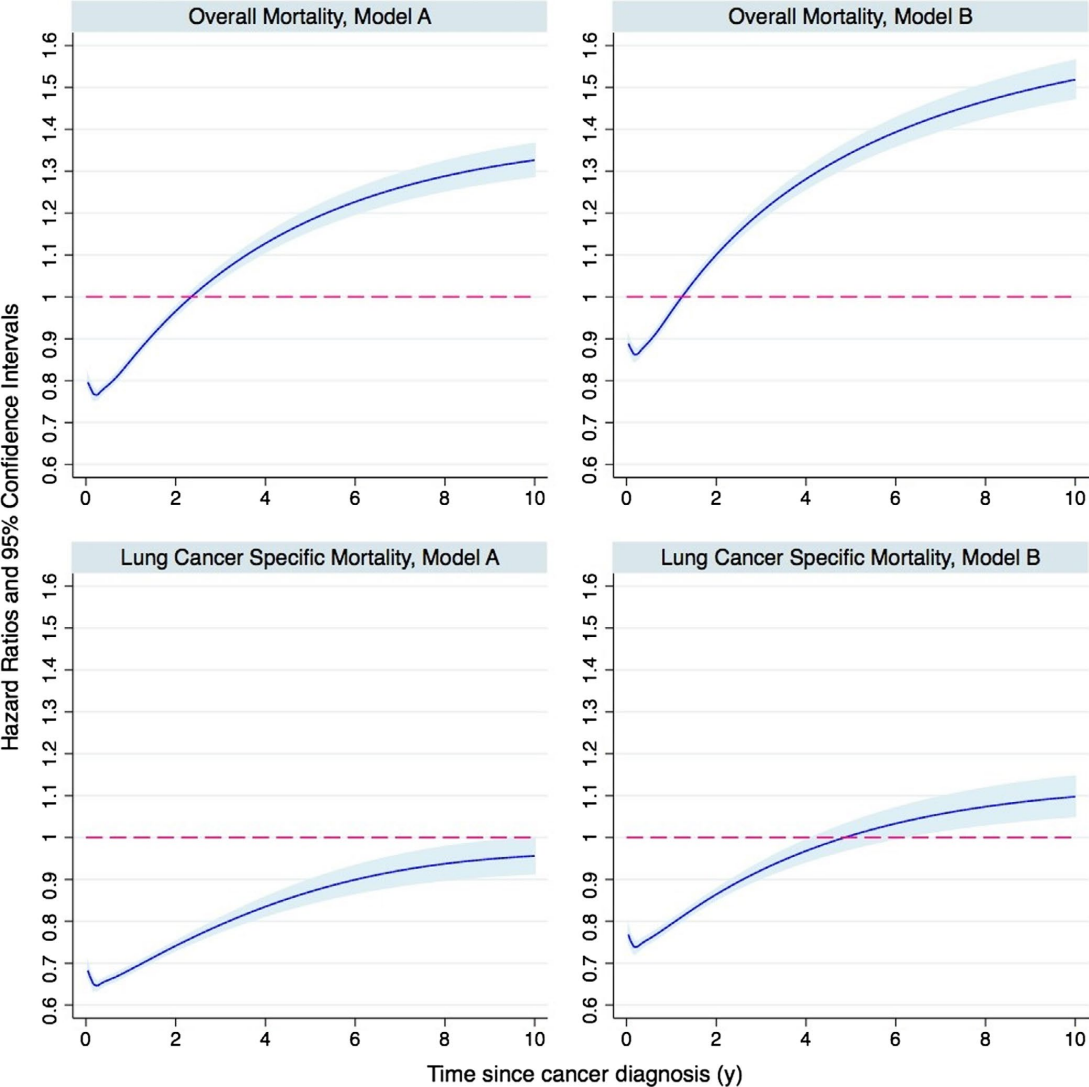
Hazards ratios (HRs) of overall and lung cancer‐specific mortality among patients with second primary lung cancer from cancer diagnosis to 10 years afterwards, compared to patients with first primary lung cancer: a population‐based cohort study in US, 1988‐2014. HRs were estimated from flexible parametric survival models, allowing the effect of second primary lung cancer to vary over time. A spline with 5 df (four intermediate knots and two knots at each boundary, placed at quintiles of distribution of events) was used for the baseline rate, while 3 df was used for the time‐varying effect. HRs in model A were adjusted for age and calendar period at diagnosis, sex, race, cohabitation status, and percentile of high‐school education and cost of living in county of residence. HRs in model B were additionally adjusted for tumor stage, histology, tumor grade, and treatment modalities

In the first 1 and 2 years after diagnosis, patients with lung‐2 had decreased risk of overall mortality when adjusting for tumor and clinical characteristics (Figure 2). However, the risk of overall mortality increased thereafter through 10 years after diagnosis of lung cancer. Regarding lung cancer‐specific mortality, lung‐2 patients had consistently decreased risk until 10 years after diagnosis. However, when adjusting for tumor and clinical characteristics, the risk of lung cancer‐specific mortality started to increase from 5 years after diagnosis. Of note, even if all deaths due to other cancers were attributed to lung cancer, lung‐2 patients still had lower risk of any cancer‐specific mortality during the first year after diagnosis (Figure S3).

To provide practical estimates, we also calculated the HRs over time by separating follow‐up into three periods (0 to <1 year, 1 to <5 years, and 5 to 10 years after diagnosis) in Table 1, which largely corroborates the temporal pattern in Figure 2. The association with lung cancer‐specific mortality was further confirmed by considering competing risks, such as dying from cancers other than lung and noncancer causes (Table S3).

**Table 1:** Hazard ratios (HRs) of overall and lung cancer‐specific mortality among patients with second primary lung cancer, by time since diagnosis, compared to patients with first primary lung cancer: a population‐based cohort study in US, 1988‐2014

	No. of patients	From 0 to < 1 y after diagnosis		From 1 to < 5 y after diagnosis		From 5 to 10 y of follow‐up after diagnosis	
		N (IR)	HR (95% CI)^a^	N (IR)	HR (95% CI)^a^	N (IR)	HR (95% CI)^a^
Overall mortality							
First primary lung cancer	679 541	383 208 (99.9)	1.00	135 513 (29.3)	1.00	16 821 (9.8)	1.00
Second primary lung cancer	85 758	44 288 (84.1)	0.91 (0.91‐0.92)	20 073 (29.4)	1.10 (1.08‐1.12)	3133 (14.6)	1.32 (1.27‐1.37)
Lung cancer‐specific mortality							
First primary lung cancer	679 541	325 633 (84.9)	1.00	111 348 (24.1)	1.00	8147 (4.7)	1.00
Second primary lung cancer	85 758	31 247 (59.3)	0.77 (0.76‐0.78)	12 485 (18.3)	0.87 (0.86‐0.89)	1158 (5.4)	1.10 (1.03‐1.17)

Note

Abbreviations: CI, confidence interval; IR, incidence rate per 100 person‐years; N, number of deaths.

a HRs were adjusted for age and calendar period at diagnosis, sex, race, cohabitation status, percentile of cost of living and high‐school education in county of residence, tumor stage, histology, tumor grade, surgery, radiation therapy, and chemotherapy.

### 3.3 Factors of first malignancy that modified the risk of mortality

Regarding lung cancer‐specific mortality, the same temporal pattern was found for all lung‐2 patients, except for patients with a history of breast and uterine cancers, who had noninferior mortality, but the monotonic increase of HR over time is similar to other cancers (Table 2). The risk reduction of lung cancer‐specific mortality during first 5 years, particularly within first year, after diagnosis was greater among lung‐2 patients with regional or distant stage as first malignancy. The earlier lung‐2 patients were diagnosed from the first malignancy, the lower the lung cancer‐specific mortality.

**Table 2:** Hazard ratios (HRs) of lung cancer‐specific mortality among patients with second primary lung cancer, by characteristics of the first malignancy, compared to patients with first primary lung cancer: a population‐based cohort study in US, 1988‐2014

	No. (%) of patients	From 0 to < 1 y after diagnosis		From 1 to < 5 y after diagnosis		From 5 to 10 y of follow‐up after diagnosis	
		N (IR)	HR (95% CI)^a^	N (IR)	HR (95% CI)^a^	N (IR)	HR (95% CI)^a^
By sites of first malignancy							
Prostate	20 661 (24.1)	8907 (75.0)	0.79 (0.78‐0.81)	3092 (22.5)	0.90 (0.87‐0.93)	255 (6.3)	1.11 (0.98‐1.27)
Breast	13 678 (15.9)	3989 (42.8)	0.68 (0.66‐0.70)	1942 (13.6)	0.75 (0.72‐0.79)	222 (4.4)	0.96 (0.84‐1.09)
Colon and rectum	9741 (11.4)	3412 (54.8)	0.72 (0.70‐0.75)	1441 (17.0)	0.83 (0.79‐0.87)	142 (5.2)	1.00 (0.85‐1.18)
Urinary bladder	8486 (9.9)	3592 (71.6)	0.83 (0.80‐0.86)	1318 (22.5)	0.95 (0.90‐1.00)	123 (7.2)	1.36 (1.14‐1.63)
Blood	6597 (7.7)	2343 (59.9)	0.84 (0.81‐0.88)	900 (18.6)	1.00 (0.93‐1.07)	68 (5.3)	1.13 (0.89‐1.43)
Larynx	3779 (4.4)	1340 (56.4)	0.80 (0.76‐0.84)	560 (20.6)	0.96 (0.88‐1.04)	50 (6.2)	1.38 (1.05‐1.83)
Skin	3108 (3.6)	1200 (61.8)	0.79 (0.74‐0.83)	482 (17.7)	0.89 (0.82‐0.98)	51 (5.4)	1.25 (0.95‐1.65)
Kidney	2565 (3.0)	803 (47.5)	0.69 (0.64‐0.74)	343 (14.2)	0.75 (0.68‐0.84)	35 (4.6)	1.06 (0.76‐1.48)
Uterus	2292 (2.7)	799 (54.3)	0.75 (0.70‐0.80)	348 (16.8)	0.85 (0.76‐0.94)	38 (4.9)	0.97 (0.70‐1.33)
Thyroid	1231 (1.4)	360 (43.2)	0.67 (0.60‐0.74)	191 (14.9)	0.79 (0.68‐0.91)	20 (4.6)	1.00 (0.65‐1.56)
Others	13 620 (15.9)	4502 (53.9)	0.78 (0.76‐0.80)	1868 (18.9)	0.92 (0.88‐0.96)	154 (5.4)	1.20 (1.02‐1.41)
By tumor stage of first malignancy							
Localized	50 465 (58.8)	19 277 (61.3)	0.80 (0.79‐0.81)	7776 (18.8)	0.88 (0.86‐0.90)	752 (5.7)	1.14 (1.05‐1.23)
Regional	15 626 (18.2)	4801 (47.7)	0.69 (0.67‐0.71)	2121 (15.8)	0.82 (0.78‐0.85)	190 (4.4)	0.97 (0.84‐1.12)
Distant	5653 (6.6)	1802 (56.5)	0.69 (0.66‐0.73)	621 (18.7)	0.86 (0.80‐0.93)	35 (4.6)	1.00 (0.71‐1.39)
Unstaged	14 014 (16.3)	5367 (64.6)	0.78 (0.76‐0.81)	1967 (19.6)	0.90 (0.86‐0.94)	181 (6.0)	1.15 (0.99‐1.34)
By time elapsed from first malignancy							
0 to < 2 y	25 925 (30.2)	7334 (43.0)	0.63 (0.62‐0.65)	3613 (15.0)	0.78 (0.76‐0.81)	402 (4.9)	1.07 (0.97‐1.19)
2 to < 5 y	21 484 (25.1)	7953 (58.9)	0.78 (0.76‐0.80)	3253 (18.7)	0.88 (0.85‐0.91)	305 (5.4)	1.09 (0.97‐1.22)
5 to < 10 y	21 268 (24.8)	8621 (67.9)	0.84 (0.83‐0.86)	3174 (20.4)	0.93 (0.90‐0.96)	249 (5.7)	1.11 (0.98‐1.26)
≥10 y	17 081 (19.9)	7339 (75.2)	0.88 (0.86‐0.90)	2445 (22.0)	0.94 (0.90‐0.98)	202 (6.2)	1.18 (1.03‐1.36)

Note:

Abbreviations: CI, confidence interval; IR, incidence rate per 100 person‐years; N, number of deaths; y, years.

a HR was adjusted for were adjusted for age and calendar period at diagnosis, sex, race, cohabitation status, percentile of cost of living and high‐school education in county of residence, tumor stage, histology, tumor grade, surgery, radiation therapy, and chemotherapy.

In terms of overall mortality, the same temporal pattern was found for all lung‐2 patients, except for patients with previous hematopoietic and lymphatic malignancies, who had increased risk of overall mortality at all time points (Table 3). More advanced stage of the first malignancy was associated with poorer overall mortality throughout follow‐up. Time from first primary did not clearly modulate the association with overall mortality.

**Table 3:** Hazard ratios (HRs) of overall mortality among patients with second primary lung cancer, by characteristics of the first malignancy, compared to patients with first primary lung cancer: a population‐based cohort study in US

	No. (%) of patients	From 0 to < 1 y after diagnosis		From 1 to < 5y after diagnosis		From 5 to 10 y of follow‐up after diagnosis	
		N (IR)	HR (95% CI)^a^	N (IR)	HR (95% CI)^a^	N (IR)	HR (95% CI)^a^
By sites of first malignancy							
Prostate	20 661 (24.1)	11 830 (99.6)	0.88 (0.86‐0.90)	4509 (32.8)	1.02 (0.99‐1.05)	693 (17.2)	1.26 (1.17‐1.36)
Breast	13 678 (15.9)	5800 (62.2)	0.83 (0.81‐0.85)	3340 (23.4)	1.03 (0.99‐1.06)	620 (12.2)	1.28 (1.18‐1.39)
Colon and rectum	9741 (11.4)	4849 (77.9)	0.85 (0.83‐0.88)	2496 (29.5)	1.10 (1.06‐1.15)	417 (15.3)	1.27 (1.16‐1.40)
Urinary bladder	8486 (9.9)	4763 (94.9)	0.92 (0.89‐0.95)	1930 (32.9)	1.09 (1.04‐1.14)	287 (16.8)	1.32 (1.17‐1.48)
Blood	6597 (7.7)	3532 (90.3)	1.06 (1.03‐1.10)	1492 (30.9)	1.28 (1.22‐1.35)	224 (17.4)	1.66 (1.46‐1.90)
Larynx	3779 (4.4)	2014 (84.8)	1.01 (0.96‐1.05)	1007 (37.0)	1.35 (1.26‐1.43)	149 (18.5)	1.72 (1.46‐2.02)
Skin	3108 (3.6)	1547 (79.7)	0.85 (0.81‐0.90)	681 (25.0)	1.00 (0.92‐1.07)	103 (10.9)	1.09 (0.90‐1.32)
Kidney	2565 (3.0)	1159 (68.6)	0.83 (0.78‐0.88)	582 (24.1)	0.99 (0.91‐1.08)	102 (13.4)	1.35 (1.11‐1.64)
Uterus	2292 (2.7)	1102 (74.9)	0.87 (0.82‐0.92)	526 (25.4)	1.01 (0.93‐1.10)	91 (11.8)	1.10 (0.89‐1.35)
Thyroid	1231 (1.4)	492 (59.0)	0.77 (0.71‐0.84)	262 (20.4)	0.88 (0.78‐0.99)	41 (9.4)	1.03 (0.76‐1.40)
Others	13 620 (15.9)	7200 (86.2)	1.05 (1.02‐1.07)	3248 (32.9)	1.27 (1.22‐1.31)	406 (14.3)	1.47 (1.33‐1.62)
By tumor stage of first malignancy							
Localized	50 465 (58.8)	25 576 (81.3)	0.89 (0.88‐0.90)	11 576 (27.9)	1.03 (1.01‐1.05)	1868 (14.1)	1.25 (1.19‐1.31)
Regional	15 626 (18.2)	7629 (75.8)	0.91 (0.89‐0.93)	3975 (29.6)	1.20 (1.16‐1.24)	585 (13.5)	1.37 (1.26‐1.48)
Distant	5653 (6.6)	3278 (102.8)	1.05 (1.02‐1.09)	1259 (38.0)	1.38 (1.31‐1.46)	139 (18.3)	1.87 (1.58‐2.21)
Unstaged	14 014 (16.3)	7805 (93.9)	0.95 (0.93‐0.97)	3263 (32.5)	1.15 (1.11‐1.19)	541 (17.8)	1.44 (1.32‐1.57)
By time elapsed from first malignancy							
0 to < 2 y	25 925 (30.2)	12 295 (72.0)	0.88 (0.86‐0.90)	6681 (27.7)	1.13 (1.10‐1.16)	1132 (13.8)	1.35 (1.27‐1.43)
2 to < 5 y	21 484 (25.1)	11 107 (82.3)	0.91 (0.89‐0.93)	5201 (29.8)	1.11 (1.08‐1.14)	822 (14.7)	1.31 (1.22‐1.40)
5 to < 10 y	21 268 (24.8)	11 462 (90.3)	0.94 (0.92‐0.96)	4718 (30.3)	1.09 (1.06‐1.12)	678 (15.7)	1.34 (1.24‐1.45)
≥10 y	17 081 (19.9)	9424 (96.6)	0.94 (0.92‐0.96)	3473 (31.3)	1.05 (1.02‐1.09)	501 (15.4)	1.27 (1.16‐1.39)

Note:

Abbreviations: CI, confidence interval; IR, incidence rate per 100 person‐years; N, number of deaths; y, years.

a HR was adjusted for were adjusted for age and calendar period at diagnosis, sex, race, cohabitation status, percentile of cost of living and high‐school education in county of residence, tumor stage, histology, tumor grade, surgery, radiation therapy, and chemotherapy.

### 3.4 Factors of lung cancer that modified the risk of mortality

When comparing lung‐2 to lung‐1, smaller HRs of lung cancer‐specific mortality were found from diagnosis to 10 years afterwards among women and patients not receiving surgery (Table S4). Men are consistently associated with worse lung cancer‐specific mortality. Larger HRs of overall mortality were observed among patients with localized stage and those who received surgery (Table S5). Other factors of lung‐2 including race did not clearly modify the association with mortality risks.

## 4 DISCUSSION

Leveraging a large population‐based cohort, to the best of our knowledge, this is the first study to comprehensively investigate the prognosis of lung cancer as the second primary malignancy (lung‐2) revealing a distinct, time‐varying disease course. Our findings suggest that lung cancer‐specific survival of lung‐2 is not necessarily inferior to lung‐1, particularly during the first 5 years after diagnosis. Such association is to some extent explained by, yet independent of, a range of important prognostic factors, including tumor and clinical characteristics. The reduced risk of overall mortality in lung‐2 is restricted to the first year after diagnosis, whereas the long‐term overall survival is in fact compromised. As lung‐2 is increasingly common in recent years, our results may provide timely guidance for clinical evaluations and decisions.

Our findings do not suggest that lung‐2 generally is more aggressive than lung‐1, although some evidence indicated that second primary cancer may be biologically different from the first one. A study of lung and breast cancer in irradiated Hodgkin lymphoma survivors showed that they had higher microsatellite alterations than their de novo counterpart.^10^ Breast cancer in this setting also demonstrated higher proliferative index.^11^ In our study, we showed that patients with lung‐2 had decreased risk of lung cancer‐specific mortality, particularly during the first 5 years after diagnosis and in patients with a previous breast or uterine cancer. This is in line with an earlier report that lung‐2 that developed in breast cancer survivors had better lung cancer‐specific survival.^5^ Studies showed that the risk of second lung cancer arises mostly 10 years after the first primary cancer.^12^, ^13^, ^14^, ^15^, ^16^ Of note, the survival benefits are somewhat attenuated among lung‐2 diagnosed 10 years after the first malignancy. Because the second primary attributed to treatment of the first primary is not common (6% among breast cancer survivors),^17^ it is, however, less likely that the presumable biological difference of lung‐2 due to treatment may substantially drive the entire association toward unfavorable survival (if any).

Given the lower lung cancer‐specific mortality in the first 5 years after diagnosis, our study argues against conservative management of lung‐2 simply because it is a second primary malignancy. One might speculate that patients with lung‐2 would receive more conservative treatment. As a matter of fact, our study showed that lung‐2 patients are managed more aggressively in the real world—they are 20% more likely to receive surgical treatment with adjustment for tumor characteristics. Because the median time to lung‐2 diagnosis is about 5 years after the first primary, it is plausible that many of lung‐2 are diagnosed during the routine surveillance of the first malignancy (ie, better access to health care) and are detected by “screening.” For instance, we observed the greatest risk reduction of lung cancer‐specific mortality among patients with lung‐2 diagnosed within 2 years after first malignancy. It is further supported by our findings that lung‐2 is more likely to be diagnosed at early stage and with a smaller size. However, the associations remain robust after fine adjustment for tumor characteristics and cancer treatment, suggesting that the favorable cancer‐specific survival among lung‐2 is independent of above prognosticators.

The time‐varying disease course of patients with lung‐2 has not been revealed previously. The favorable overall survival in lung‐2 was restricted to the first year after diagnosis. In the long run, patients with lung‐2 had worse overall survival. It is plausible that the progression of first malignancy contributed to the increased risks of overall mortality from 1 year after diagnosis onward. It is supported by the fact that highest increased risk of overall mortality, but not lung cancer‐specific mortality among lung‐2 patients with first malignancy diagnosed at distant stage.

Previous studies that investigated lung‐2 in cancer survivors of specific cancer sites have yielded seemingly inconsistent results. Overall survival of non‐small cell lung cancer in Hodgkin lymphoma and chronic lymphocytic leukemia survivors has been reported to be worse than their de novo counterparts,^4^, ^18^ but not in breast cancer survivors.^5^ These seemingly contrary findings to ours are explained by our approach of examining sites of first malignancies individually and disentangling temporal patterns. Lung‐2 patients with hematopoietic and lymphatic malignancy as the first primary stood out as the only primary malignancies that had worse overall survival during follow‐up. For the remaining first malignancies, including breast cancer, lung‐2 patients did not have elevated risk of overall mortality within the first year of diagnosis. Our findings suggest that factors other than lung‐2, such as the first primary cancer may play a significant role in the overall mortality of lung‐2 patients.

The major strength of our study is the large‐scale population‐based prospective cohort of patients with primary lung cancer, which assures minimal common biases including selection and surveillance biases. Our comprehensive analyses on the temporal pattern of mortality risk and characteristics of first malignancy helped explain conflicting results in previous literatures. Our study has several limitations. First, findings could be in part explained by competing risk, yet reassuringly, analysis with competing risk model resulted in even stronger associations. Second, the cause of death may be recorded incorrectly, but a validation study showed fairly good agreement rate for lung cancer‐specific survival.^19^ Also, our additional analysis on any cancer‐specific mortality showed that even attributing all deaths of other cancers to lung cancer, lung‐2 patients remain at a reduced risk of any cancer‐specific mortality during the first year after diagnosis. Third, a few factors that associated with survival are not recorded, including tobacco use and comorbidities, although such factors are more influential to overall, instead of cancer‐specific, mortality. Finally, our study did not include lung‐2 with lung cancer as its first primary, due to the challenge of disentangling lung‐2 from pulmonary metastases.

In conclusion, patients with second primary lung cancer have favorable lung cancer‐specific survival during the first 5 years after diagnosis, but not afterwards, compared with patients with first primary lung cancer. Such association is independent of tumor characteristics and cancer treatment. Our findings suggest that the disease course of lung‐2 is not more aggressive than lung‐1. There is not enough reason opting for conservative care simply because of history of first malignancy. The compromised overall survival of lung‐2 patients in the longer term suggests the importance of active management and surveillance of first malignancy for optimizing overall survival.

## CONFLICT OF INTEREST

The authors have declared no conflict of interest.

## AUTHOR CONTRIBUTIONS

LD, BL, and DL conceptualized the study. LD and DL designed the study. LD and DL collected and analyzed the data. HS analyzed data. All authors discussed and interpreted the results. LD and DL drafted the manuscript. All authors significantly revised and gave final approval of the version submitted.
