# Supplementary figures and images for: Mortality of lung cancer as a second primary malignancy: A population‐based cohort study
Source: Cancer Med. 2019 Apr 16;8(6):3269–77. doi: 10.1002/cam4.2172 (PMC6558593; doi:10.1002/cam4.2172)

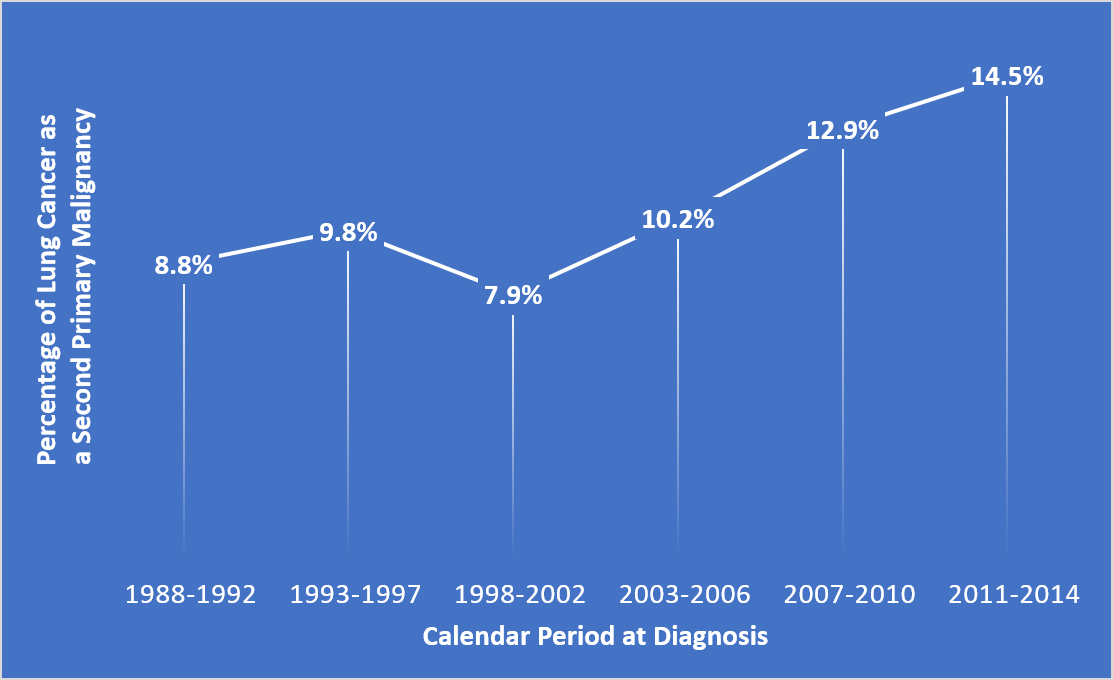

Supplement: Supplementary file 1 [file CAM4-8-3269-s001.tif]

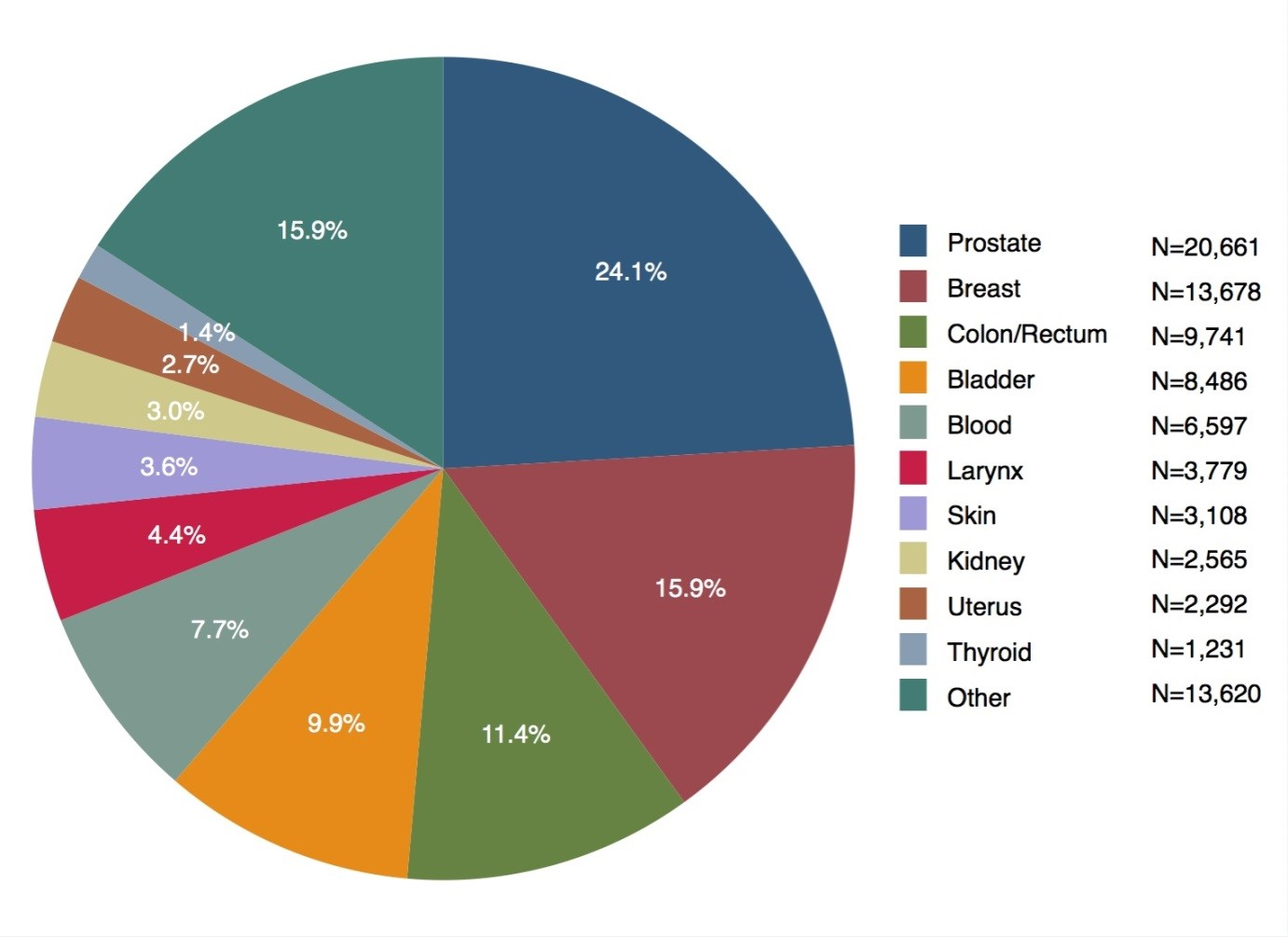

Supplement: Supplementary file 2 [file CAM4-8-3269-s002.tif]

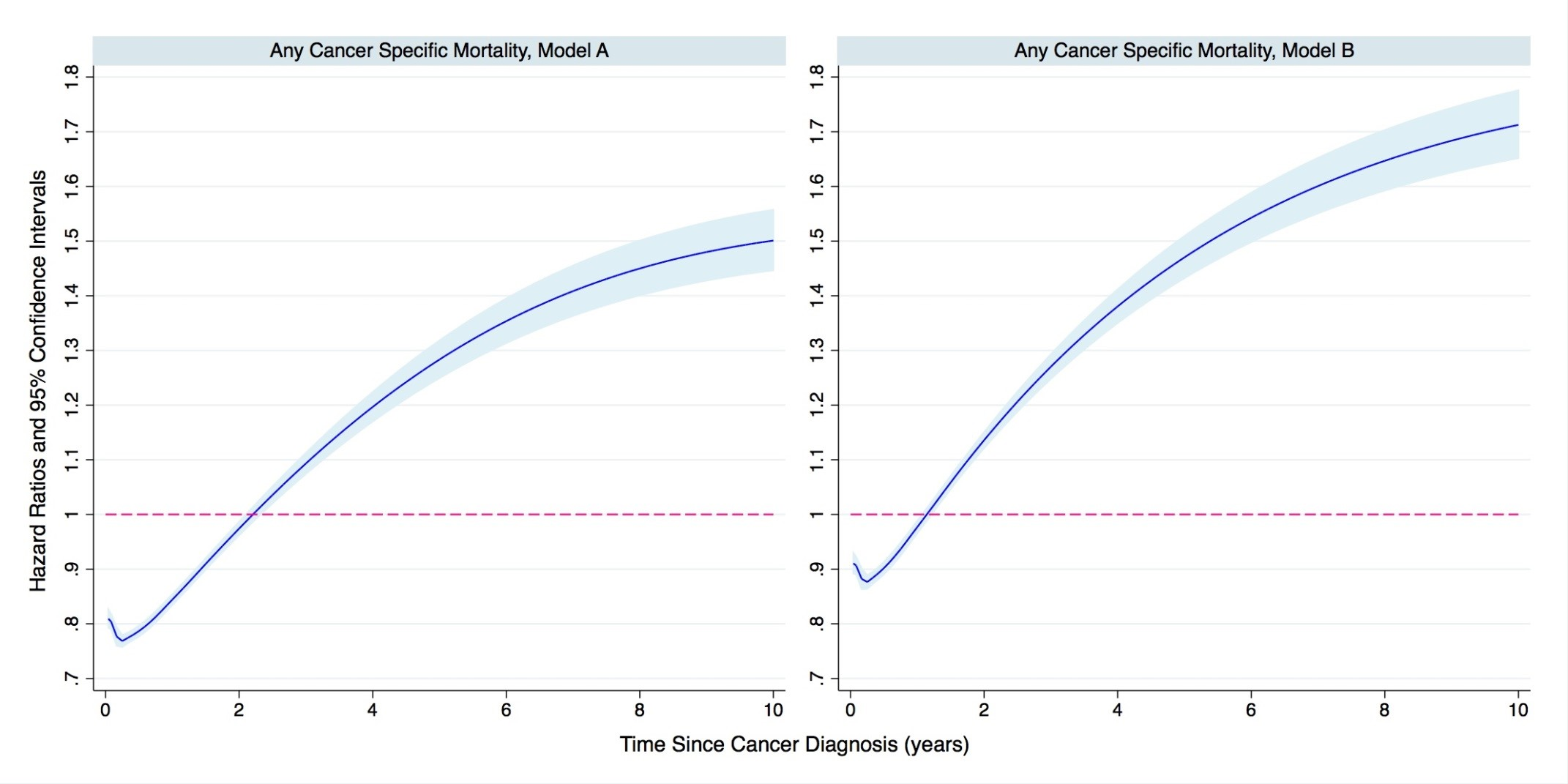

Supplement: Supplementary file 3 [file CAM4-8-3269-s003.tif]
