# Supplementary material for: Mortality of lung cancer as a second primary malignancy: A population‐based cohort study
Source: Cancer Med. 2019 Apr 16;8(6):3269–77. doi: 10.1002/cam4.2172 (PMC6558593; doi:10.1002/cam4.2172)
Supplement: Supplementary file 4 [file CAM4-8-3269-s004.docx]

**Table S1 Baseline characteristics of patients with first and second primary lung cancers: a population-based cohort study in US, 1988-2014.**

|  | **Patients with first primary lung cancer** | | **Patients with second primary lung cancer** | |
| --- | --- | --- | --- | --- |
|  | ***N*** | **%** | ***N*** | **%** |
| Total number | 679,541 | - | 85,758 | - |
| Age at diagnosis, years |  |  |  |  |
| 18-44 | 15,866 | 2.3 | 572 | 0.7 |
| 45-54 | 68,964 | 10.1 | 3,558 | 4.1 |
| 55-64 | 161,234 | 23.7 | 13,667 | 15.9 |
| 65-74 | 224,356 | 33.0 | 30,202 | 35.2 |
| 75-84 | 166,311 | 24.5 | 29,918 | 34.9 |
| ≥85 | 42,810 | 6.3 | 7,841 | 9.1 |
| Sex |  |  |  |  |
| Men | 367,647 | 54.1 | 51,177 | 59.7 |
| Women | 311,894 | 45.9 | 34,581 | 40.3 |
| Race |  |  |  |  |
| White | 551,851 | 81.2 | 71,989 | 83.9 |
| Black | 81,942 | 12.1 | 9,588 | 11.2 |
| Asian | 41,252 | 6.1 | 3,875 | 4.5 |
| Other | 4,496 | 0.7 | 306 | 0.4 |
| Cohabitation status |  |  |  |  |
| Non-cohabitated | 304,872 | 44.9 | 35,152 | 41.0 |
| Cohabitated | 347,431 | 51.1 | 47,005 | 54.8 |
| Unknown | 27,238 | 4.0 | 3,601 | 4.2 |
| % of High-school education in the county of residence |  |  |  |  |
| Lowest tertile | 135,320 | 19.9 | 15,583 | 18.2 |
| Middle tertile | 246,281 | 36.2 | 27,479 | 32.0 |
| Highest tertile | 297,940 | 43.8 | 42,696 | 49.8 |
| Cost of living adjusted median household income in the county of residence |  |  |  |  |
| Lowest tertile | 62,739 | 9.2 | 5,880 | 6.9 |
| Middle tertile | 107,912 | 15.9 | 12,665 | 14.8 |
| Highest tertile | 508,890 | 74.9 | 67,213 | 78.4 |
| Calendar period at diagnosis |  |  |  |  |
| 1988-1992 | 46,193 | 6.8 | 4,458 | 5.2 |
| 1993-1997 | 63,266 | 9.3 | 6,869 | 8.0 |
| 1998-2002 | 128,628 | 18.9 | 11,031 | 12.9 |
| 2003-2006 | 142,685 | 21.0 | 16,145 | 18.8 |
| 2007-2010 | 150,985 | 22.2 | 22,264 | 26.0 |
| 2011-2014 | 147,784 | 21.7 | 24,991 | 29.1 |
| Tumor stage^*^ |  |  |  |  |
| Localized | 115,704 | 17.0 | 22,117 | 25.8 |
| Regional | 165,322 | 24.3 | 21,151 | 24.7 |
| Distant | 362,823 | 53.4 | 38,067 | 44.4 |
| Unknown | 35,692 | 5.3 | 4,423 | 5.2 |
| Histology |  |  |  |  |
| Small cell | 93,988 | 13.8 | 9,130 | 10.6 |
| Non-small cell |  |  |  |  |
| Squamous cell | 136,268 | 20.1 | 19,731 | 23.0 |
| Adenocarcinoma | 230,393 | 33.9 | 31,304 | 36.5 |
| Adenosquamous cell | 7,425 | 1.1 | 1,034 | 1.2 |
| Large cell | 23,672 | 3.5 | 2,203 | 2.6 |
| Non-small-cell, NOS | 65,802 | 9.7 | 7,612 | 8.9 |
| Other | 121,993 | 18.0 | 14,744 | 17.2 |
| Tumor grade |  |  |  |  |
| Well differentiated | 27,488 | 4.0 | 4,901 | 5.7 |
| Moderately differentiated | 90,642 | 13.3 | 14,352 | 16.7 |
| Poorly differentiated | 170,569 | 25.1 | 21,209 | 24.7 |
| Undifferentiated | 46,734 | 6.9 | 4,333 | 5.1 |
| Unknown | 344,108 | 50.6 | 40,963 | 47.8 |
| Tumor size |  |  |  |  |
| 0 to 3 cm | 185,898 | 27.4 | 30,718 | 35.8 |
| >3 to 5 cm | 140,781 | 20.7 | 17,450 | 20.3 |
| >5 to 7 cm | 77,775 | 11.4 | 8,418 | 9.8 |
| >7 cm | 64,602 | 9.5 | 6,095 | 7.1 |
| Unknown | 210,485 | 31.0 | 23,077 | 26.9 |
| Surgery^†^ |  |  |  |  |
| No | 447,161 | 78.4 | 53,893 | 72.4 |
| Yes | 116,743 | 20.5 | 20,060 | 27.0 |
| Unknown | 6,178 | 1.1 | 478 | 0.6 |
| Radiation therapy |  |  |  |  |
| No/Unknown | 394,796 | 58.1 | 54,540 | 63.6 |
| Yes | 284,745 | 41.9 | 31,218 | 36.4 |
| Chemotherapy |  |  |  |  |
| No/Unknown | 408,242 | 60.1 | 56,918 | 66.4 |
| Yes | 271,299 | 39.9 | 28,840 | 33.6 |

*N*, number; IR, incidence rate, per 100 person-years; NOS, not otherwise specified.

^*^ Tumor staging was based on SEER historic staging, i.e. localized, regional, and distant to ensure the consistency across the long range of the study period, as TNM staging system has been evolving during the study period.

^†^ Information on surgery was available from 1998 onward.

**Table S2. Associations of second primary lung cancer with tumor and clinical characteristics, compared to patients with first primary lung cancer: a population-based cohort study in US.**

|  | **First primary lung cancer** | **Second primary lung cancer** |
| --- | --- | --- |
|  | **OR/RRR** | **OR/RRR (95% CI)** ^*^ |
| **Tumor Characteristics** |  |  |
| Localized tumor | 1.00 | 1.64 (1.61-1.67) |
| Tumor size ≤3 cm | 1.00 | 1.51 (1.49-1.54) |
| Histology |  |  |
| Small cell | 1.00 | 0.81 (0.79-0.83) |
| Squamous cell | 1.00 | 1.08 (1.06-1.10) |
| Tumor grade |  |  |
| Well differentiated | 1.00 | 1.39 (1.35-1.44) |
| Moderately differentiated | 1.00 | 1.24 (1.21-1.27) |
|  |  |  |
|  | **OR** | **OR (95% CI)** ^†^ |
| **Treatment Modalities** |  |  |
| Radiation therapy | 1.00 | 0.93 (0.92-0.95) |
| Chemotherapy | 1.00 | 1.00 (0.98-1.02) |
| Surgery | 1.00 | 1.20 (1.17-1.23) |

HR, hazard ratio; OR, odds ratio; RRR, relative risk ratio; CI, confidence interval.

^*^ The models were adjusted for age and calendar period at diagnosis, sex, race, cohabitation status, and percentile of cost of living and high-school education in county of residence. Histology and tumor grade were presented by relative risk ratio; the rest were presented with odds ratio.

^†^ The models were adjusted for age and calendar period at diagnosis, sex, race, cohabitation status, percentile of cost of living and high-school education in county of residence, tumor stage, histology, and tumor grade.

**Table S3. Associations of second primary lung cancer with lung cancer specific mortality, compared to patients with first primary lung cancer, using competing risk model: a population-based cohort study in US.**

|  | **No. (%) of patients** | **From 0 to <1 year after diagnosis** | | **From 1 year to < 5 years after diagnosis** | | **From 5 years to 10 years of follow-up after diagnosis** | |
| --- | --- | --- | --- | --- | --- | --- | --- |
|  |  | **N (IR)** | **HR (95% CI) ^*^** | **N (IR)** | **HR (95% CI)^*^** | **N (IR)** | **HR (95% CI)^*^** |
| **Lung cancer specific mortality** |  |  |  |  |  |  |  |
| First primary lung cancer | 679,541 | 325,633 (84.9) | 1.00 | 111,348 (24.1) | 1.00 | 8,147 (4.7) | 1.00 |
| Second primary lung cancer | 85,758 | 31,247 (59.3) | 0.73 (0.72-0.74) | 12,485 (18.3) | 0.80 (0.78-0.81) | 1,158 (5.4) | 1.04 (0.98-1.11) |

N, number of deaths; IR, incidence rate per 100 person-years; CI, confidence interval.

^*^ HR was adjusted for were adjusted for age and calendar period at diagnosis, sex, race, cohabitation status, percentile of cost of living and high-school education in county of residence, tumor stage, histology, tumor grade, surgery, radiation therapy, and chemotherapy.

**Table S4. Hazard ratios (HRs) of cancer specific mortality among patients with second primary lung cancer (lung-2), stratified by demographic, tumor, and clinical characteristics, compared to patients with first primary lung cancer (lung-1): a population-based cohort study in US, 1988-2014.**

|  | **From 0 to <1 year after diagnosis** | | | **From 1 year to < 5 years after diagnosis** | | | **From 5 years to 10 years after diagnosis** | | |
| --- | --- | --- | --- | --- | --- | --- | --- | --- | --- |
|  | **Lung-1** | **Lung-2** | **HR (95% CI)** | **Lung-1** | **Lung-2** | **HR (95% CI)** | **Lung-1** | **Lung-2** | **HR (95% CI)** |
|  | **N (IR)** | **N (IR)** |  | **N (IR)** | **N (IR)** |  | **N (IR)** | **N (IR)** |  |
| **Age at diagnosis** |  |  |  |  |  |  |  |  |  |
| 18-44 | 6,210 (56.4) | 125 (30.2) | 0.68 (0.57-0.82) | 3,002 (17.3) | 64 (9.6) | 0.77 (0.60-0.99) | 239 (2.3) | 7 (2.0) | 0.88 (0.41-1.89) |
| 45-54 | 30,501 (68.2) | 907 (35.7) | 0.67 (0.63-0.72) | 13,941 (23.4) | 482 (12.3) | 0.73 (0.67-0.80) | 972 (3.4) | 60 (3.6) | 1.22 (0.93-1.58) |
| 55-64 | 72,612 (72.9) | 3,985 (42.1) | 0.72 (0.70-0.75) | 29,682 (23.4) | 2,161 (15.4) | 0.85 (0.81-0.89) | 2,270 (4.3) | 217 (3.9) | 0.98 (0.85-1.13) |
| 65-74 | 104,458 (80.3) | 10,195 (51.5) | 0.77 (0.75-0.79) | 37,106 (23.3) | 4,813 (17.8) | 0.90 (0.87-0.93) | 2,979 (5.3) | 495 (5.6) | 1.13 (1.03-1.25) |
| 75-84 | 86,713 (103.7) | 12,308 (71.7) | 0.80 (0.78-0.81) | 23,389 (26.6) | 4,140 (21.1) | 0.88 (0.86-0.91) | 1,531 (6.6) | 349 (7.6) | 1.15 (1.03-1.30) |
| ≥85 | 25,139 (149.1) | 3,727 (102.1) | 0.80 (0.77-0.83) | 4,228 (34.8) | 825 (27.5) | 0.86 (0.80-0.93) | 156 (9.4) | 30 (8.1) | 0.86 (0.57-1.29) |
|  |  |  |  |  |  |  |  |  |  |
| **Sex** |  |  |  |  |  |  |  |  |  |
| Men | 186,383 (93.0) | 20,346 (67.3) | 0.79 (0.77-0.80) | 57,790 (26.6) | 7,445 (21.2) | 0.90 (0.88-0.92) | 3,684 (4.8) | 630 (6.3) | 1.19 (1.09-1.30) |
| Women | 139,250 (75.1) | 10,901 (47.9) | 0.75 (0.73-0.76) | 53,558 (21.8) | 5,040 (15.2) | 0.84 (0.81-0.86) | 4,463 (4.7) | 528 (4.6) | 1.00 (0.91-1.10) |
|  |  |  |  |  |  |  |  |  |  |
| **Race** |  |  |  |  |  |  |  |  |  |
| White | 265,892 (85.0) | 26,498 (59.6) | 0.78 (0.77-0.79) | 89,282 (23.5) | 10,495 (18.2) | 0.88 (0.87-0.90) | 6,543 (4.6) | 970 (5.3) | 1.11 (1.04-1.19) |
| Black | 40,057 (87.7) | 3,403 (58.3) | 0.71 (0.69-0.74) | 13,769 (28.3) | 1,351 (19.9) | 0.78 (0.74-0.83) | 912 (5.5) | 124 (6.3) | 1.06 (0.87-1.28) |
| Asian | 17,605 (70.4) | 1,252 (49.5) | 0.80 (0.75-0.84) | 7,636 (24.3) | 592 (16.8) | 0.88 (0.81-0.96) | 651 (5.9) | 60 (5.0) | 0.88 (0.68-1.16) |
| Other | 2,079 (81.7) | 94 (48.1) | 0.72 (0.59-0.89) | 661 (22.3) | 47 (18.1) | 0.98 (0.73-1.33) | 41 (3.9) | 4 (5.2) | 1.80 (0.60-5.41) |
|  |  |  |  |  |  |  |  |  |  |
| **Cohabitation status** | | | | | | | | | |
| No | 153,831 (94.7) | 13,263 (63.8) | 0.76 (0.75-0.78) | 45,989 (25.4) | 4,803 (18.8) | 0.84 (0.82-0.87) | 3,208 (5.2) | 432 (5.9) | 1.10 (0.99-1.21) |
| Yes | 159,115 (76.3) | 16,786 (56.0) | 0.78 (0.77-0.79) | 61,267 (23.2) | 7,229 (18.1) | 0.90 (0.88-0.92) | 4,688 (4.5) | 699 (5.3) | 1.12 (1.03-1.21) |
| Unknown | 12,687 (84.4) | 1,198 (53.9) | 0.75 (0.70-0.79) | 4,092 (23.4) | 453 (16.1) | 0.80 (0.72-0.88) | 251 (4.2) | 27 (3.4) | 0.80 (0.53-1.20) |
|  |  |  |  |  |  |  |  |  |  |
| **% of High-school education in county of residence** | | | | | | | | | |
| Lowest tertile | 66,395 (89.3) | 5,681 (60.6) | 0.76 (0.74-0.78) | 21,089 (25.5) | 2,114 (18.8) | 0.86 (0.82-0.90) | 1,362 (5.0) | 168 (5.4) | 1.04 (0.89-1.23) |
| Middle tertile | 119,229 (86.0) | 9,763 (57.8) | 0.75 (0.73-0.76) | 39,947 (24.6) | 3,791 (18.0) | 0.84 (0.81-0.87) | 2,819 (5.0) | 314 (5.4) | 1.02 (0.91-1.15) |
| Highest tertile | 140,009 (80.9) | 15,803 (59.0) | 0.80 (0.78-0.81) | 50,312 (23.1) | 6,580 (18.3) | 0.90 (0.87-0.92) | 3,966 (4.5) | 676 (5.4) | 1.16 (1.07-1.26) |
|  |  |  |  |  |  |  |  |  |  |
| **Cost of living adjusted median household income in county of residence** | | | | | | | | | |
| Lowest tertile | 31,502 (91.4) | 2,149 (60.7) | 0.74 (0.70-0.77) | 9,802 (26.6) | 838 (21.5) | 0.91 (0.85-0.98) | 632 (5.1) | 50 (5.0) | 0.95 (0.71-1.27) |
| Middle tertile | 52,604 (88.5) | 4,557 (59.5) | 0.76 (0.73-0.78) | 16,415 (25.3) | 1,671 (19.0) | 0.86 (0.82-0.90) | 896 (5.1) | 90 (5.2) | 0.98 (0.79-1.22) |
| Highest tertile | 241,527 (82.7) | 24,541 (58.7) | 0.78 (0.77-0.79) | 85,131 (23.6) | 9,976 (17.9) | 0.87 (0.85-0.89) | 6,619 (4.7) | 1,018 (5.5) | 1.12 (1.05-1.20) |
|  |  |  |  |  |  |  |  |  |  |
| **Calendar period at diagnosis** | | | | | | | | | |
| 1988-1992 | 32,846 (90.9) | 2,996 (72.2) | 0.83 (0.80-0.86) | 11,273 (24.8) | 1,081 (19.1) | 0.85 (0.80-0.91) | 1,127 (4.4) | 171 (5.5) | 1.23 (1.05-1.45) |
| 1993-1997 | 32,846 (90.9) | 2,996 (72.2) | 0.83 (0.80-0.86) | 11,273 (24.8) | 1,081 (19.1) | 0.85 (0.80-0.91) | 1,127 (4.4) | 171 (5.5) | 1.23 (1.05-1.45) |
| 1998-2002 | 66,066 (89.8) | 4,321 (63.1) | 0.74 (0.71-0.76) | 23,714 (24.6) | 1,873 (19.1) | 0.84 (0.80-0.88) | 2,473 (4.7) | 287 (5.5) | 1.09 (0.96-1.23) |
| 2003-2006 | 70,927 (85.8) | 6,057 (58.7) | 0.75 (0.73-0.77) | 26,808 (23.5) | 2,862 (17.8) | 0.86 (0.83-0.90) | 2,745 (4.8) | 404 (5.0) | 1.02 (0.92-1.13) |
| 2007-2010 | 71,062 (79.7) | 7,998 (54.7) | 0.77 (0.75-0.79) | 28,235 (22.1) | 3,885 (16.4) | 0.87 (0.84-0.90) | 1,032 (5.6) | 202 (6.2) | 1.13 (0.97-1.32) |
| 2011-2014 | 32,846 (90.9) | 2,996 (72.2) | 0.83 (0.80-0.86) | 11,273 (24.8) | 1,081 (19.1) | 0.85 (0.80-0.91) | 1,127 (4.4) | 171 (5.5) | 1.23 (1.05-1.45) |
|  |  |  |  |  |  |  |  |  |  |
| **Tumor stage** |  |  |  |  |  |  |  |  |  |
| Localized | 16,920 (18.2) | 2,490 (13.5) | 0.73 (0.70-0.76) | 18,711 (9.5) | 3,303 (9.2) | 0.92 (0.88-0.95) | 2,861 (3.1) | 601 (4.6) | 1.30 (1.19-1.42) |
| Regional | 56,228 (48.0) | 6,052 (39.3) | 0.81 (0.79-0.83) | 38,664 (23.8) | 4,327 (20.9) | 0.91 (0.88-0.94) | 3,352 (5.7) | 375 (6.2) | 1.00 (0.90-1.12) |
| Distant | 234,447 (150.1) | 21,074 (126.7) | 0.78 (0.77-0.79) | 47,392 (56.3) | 4,179 (46.6) | 0.83 (0.80-0.86) | 1,505 (10.0) | 132 (9.6) | 0.84 (0.70-1.01) |
| Unknown | 18,038 (93.5) | 1,631 (62.6) | 0.69 (0.65-0.72) | 6,581 (34.1) | 676 (24.8) | 0.72 (0.66-0.78) | 429 (6.8) | 50 (6.6) | 0.81 (0.60-1.09) |
|  |  |  |  |  |  |  |  |  |  |
| **Histology** |  |  |  |  |  |  |  |  |  |
| Small cell | 54,781 (108.1) | 4,805 (101.4) | 0.84 (0.82-0.87) | 17,304 (51.6) | 1,357 (45.9) | 0.90 (0.85-0.95) | 600 (6.1) | 50 (7.3) | 1.07 (0.80-1.44) |
| Squamous cell | 59,740 (71.6) | 6,714 (51.9) | 0.79 (0.77-0.81) | 23,854 (23.2) | 3,227 (20.0) | 0.93 (0.89-0.96) | 1,663 (4.6) | 271 (5.9) | 1.24 (1.09-1.41) |
| Adenocarcinoma | 91,882 (71.2) | 9,101 (48.3) | 0.78 (0.76-0.79) | 37,739 (22.5) | 4,348 (16.3) | 0.85 (0.83-0.88) | 3,193 (5.5) | 483 (6.0) | 1.06 (0.96-1.17) |
| Other | 119,230 (97.1) | 10,627 (64.4) | 0.73 (0.72-0.75) | 32,451 (20.5) | 3,553 (15.8) | 0.85 (0.82-0.88) | 2,691 (4.0) | 354 (4.4) | 1.08 (0.96-1.20) |
|  |  |  |  |  |  |  |  |  |  |
| **Tumor size** |  |  |  |  |  |  |  |  |  |
| 0 to 3 cm | 54,670 (41.9) | 6,045 (25.9) | 0.75 (0.73-0.77) | 31,913 (14.2) | 4,647 (11.6) | 0.90 (0.88-0.93) | 3,642 (3.9) | 666 (4.9) | 1.19 (1.10-1.30) |
| >3 to 5 cm | 61,433 (70.9) | 6,315 (56.3) | 0.83 (0.81-0.86) | 26,748 (25.5) | 2,898 (21.4) | 0.90 (0.86-0.93) | 1,952 (5.3) | 227 (5.6) | 0.99 (0.87-1.14) |
| >5 to 7 cm | 40,909 (95.4) | 4,004 (86.2) | 0.85 (0.82-0.88) | 13,146 (32.3) | 1,266 (30.2) | 0.92 (0.86-0.97) | 696 (5.2) | 73 (6.5) | 1.18 (0.92-1.50) |
| >7 cm | 38,443 (121.3) | 3,284 (109.9) | 0.84 (0.81-0.87) | 9,451 (38.4) | 775 (34.8) | 0.94 (0.87-1.01) | 421 (5.8) | 45 (7.6) | 1.17 (0.85-1.60) |
| Unknown | 130,178 (138.2) | 11,599 (106.7) | 0.75 (0.74-0.76) | 30,090 (44.1) | 2,899 (34.8) | 0.81 (0.78-0.84) | 1,436 (7.2) | 147 (7.2) | 0.90 (0.76-1.07) |
|  |  |  |  |  |  |  |  |  |  |
| **Tumor grade (differentiation)** | | | | | | | | | |
| Well | 5,257 (24.6) | 667 (16.7) | 0.76 (0.70-0.82) | 3,891 (8.8) | 587 (7.2) | 0.91 (0.83-0.99) | 664 (3.4) | 134 (4.6) | 1.23 (1.02-1.49) |
| Moderate | 26,021 (39.8) | 2,975 (26.9) | 0.79 (0.76-0.82) | 16,832 (14.9) | 2,385 (12.4) | 0.93 (0.89-0.97) | 2,093 (4.7) | 323 (5.0) | 1.04 (0.92-1.17) |
| Poor | 79,932 (78.4) | 7,668 (56.0) | 0.79 (0.77-0.81) | 29,910 (23.2) | 3,450 (18.9) | 0.90 (0.87-0.93) | 2,352 (4.8) | 337 (5.8) | 1.17 (1.04-1.31) |
| Undifferentiated | 26,199 (99.8) | 2,140 (87.9) | 0.85 (0.82-0.89) | 8,777 (36.4) | 693 (30.0) | 0.93 (0.86-1.01) | 502 (5.0) | 48 (5.5) | 1.10 (0.82-1.49) |
| Unknown | 188,224 (110.1) | 17,797 (81.4) | 0.75 (0.74-0.77) | 51,938 (34.1) | 5,370 (26.5) | 0.82 (0.80-0.85) | 2,536 (5.3) | 316 (6.0) | 1.04 (0.92-1.17) |
|  |  |  |  |  |  |  |  |  |  |
| **Surgery**^*^ |  |  |  |  |  |  |  |  |  |
| No | 252,502 (114.0) | 24,300 (85.2) | 0.76 (0.75-0.77) | 71,635 (44.6) | 7,486 (34.5) | 0.81 (0.79-0.83) | 2,892 (9.8) | 312 (9.7) | 0.87 (0.78-0.98) |
| Yes | 12,817 (13.0) | 1,799 (10.3) | 0.83 (0.79-0.87) | 18,975 (8.6) | 3,057 (8.2) | 1.01 (0.97-1.05) | 3,319 (3.4) | 579 (4.3) | 1.19 (1.09-1.31) |
| Unknown | 3,805 (125.2) | 243 (100.5) | 0.76 (0.67-0.87) | 916 (37.0) | 55 (26.0) | 0.71 (0.54-0.94) | 39 (5.0) | 2 (4.8) | 0.81 (0.17-3.79) |
|  |  |  |  |  |  |  |  |  |  |
| **Radiation therapy** |  |  |  |  |  |  |  |  |  |
| No/Unknown | 182,809 (86.1) | 18,462 (55.9) | 0.75 (0.74-0.76) | 52,554 (17.1) | 6,785 (13.5) | 0.86 (0.84-0.88) | 4,867 (3.7) | 821 (4.6) | 1.12 (1.04-1.21) |
| Yes | 142,824 (82.3) | 12,785 (63.9) | 0.82 (0.80-0.83) | 58,794 (37.9) | 5,700 (31.8) | 0.89 (0.86-0.91) | 3,280 (8.2) | 337 (10.0) | 1.06 (0.94-1.19) |
|  |  |  |  |  |  |  |  |  |  |
| **Chemotherapy** |  |  |  |  |  |  |  |  |  |
| No/Unknown | 202,130 (99.9) | 20,234 (61.4) | 0.75 (0.74-0.76) | 45,912 (15.5) | 6,332 (12.7) | 0.86 (0.83-0.88) | 4,771 (3.7) | 839 (4.8) | 1.20 (1.11-1.29) |
| Yes | 123,503 (67.3) | 11,013 (54.9) | 0.84 (0.82-0.86) | 65,436 (39.2) | 6,153 (33.2) | 0.89 (0.86-0.91) | 3,376 (7.9) | 319 (8.0) | 0.91 (0.81-1.02) |

N, number of deaths; IR, incidence rate per 100 person-years; HR, hazard ratio; CI, confidence interval. HRs were adjusted for age and calendar period at diagnosis, sex, race, cohabitation status, % of high-school education in county of residence, tumor stage, histology, tumor grade, surgery, radiation therapy, and chemotherapy, if applicable.

^*^ Information on surgery was available from 1998 onward.

**Table S5. Hazard ratios (HRs) of overall mortality among patients with second primary lung cancer, stratified by demographic, tumor, and clinical characteristics, compared to patients with first primary lung cancer: a population-based cohort study in US.**

|  | **From 0 to <1 year after diagnosis** | | | **From 1 year to < 5 years after diagnosis** | | | **From 5 years to 10 years after diagnosis** | | |
| --- | --- | --- | --- | --- | --- | --- | --- | --- | --- |
|  | **First primary Lung Cancer** | **Second primary Lung Cancer** | **HR (95% CI)** | **First primary Lung Cancer** | **Second primary Lung Cancer** | **HR (95% CI)** | **First primary Lung Cancer** | **Second primary Lung Cancer** | **HR (95% CI)** |
|  | **N (IR)** | **N (IR)** |  | **N (IR)** | **N (IR)** |  | **N (IR)** | **N (IR)** |  |
| **Age at diagnosis** |  |  |  |  |  |  |  |  |  |
| 18-44 | 7,168 (65.2) | 238 (57.6) | 1.09 (0.96-1.25) | 3,427 (19.8) | 138 (20.7) | 1.41 (1.19-1.68) | 347 (3.3) | 17 (4.8) | 1.36 (0.83-2.25) |
| 45-54 | 34,603 (77.4) | 1,463 (57.6) | 0.94 (0.89-0.99) | 15,672 (26.3) | 884 (22.6) | 1.17 (1.09-1.25) | 1,540 (5.4) | 148 (8.9) | 1.84 (1.55-2.19) |
| 55-64 | 83,107 (83.4) | 5,890 (62.2) | 0.92 (0.90-0.95) | 34,469 (27.2) | 3,420 (24.4) | 1.13 (1.09-1.17) | 3,973 (7.6) | 557 (10.0) | 1.40 (1.28-1.53) |
| 65-74 | 122,449 (94.1) | 14,314 (72.4) | 0.91 (0.90-0.93) | 45,324 (28.5) | 7,498 (27.8) | 1.12 (1.09-1.14) | 6,367 (11.4) | 1,278 (14.4) | 1.32 (1.24-1.40) |
| 75-84 | 104,230 (124.6) | 17,070 (99.4) | 0.92 (0.90-0.93) | 30,527 (34.7) | 6,740 (34.3) | 1.08 (1.05-1.10) | 4,081 (17.6) | 1,018 (22.1) | 1.25 (1.17-1.34) |
| ≥85 | 31,651 (187.8) | 5,313 (145.6) | 0.91 (0.89-0.94) | 6,094 (50.1) | 1,393 (46.4) | 1.00 (0.94-1.06) | 513 (30.9) | 115 (30.9) | 1.05 (0.85-1.29) |
|  |  |  |  |  |  |  |  |  |  |
| **Sex** |  |  |  |  |  |  |  |  |  |
| Men | 219,511 (109.5) | 28,497 (94.2) | 0.92 (0.91-0.93) | 70,589 (32.5) | 11,752 (33.4) | 1.10 (1.08-1.13) | 8,022 (10.5) | 1,694 (17.0) | 1.33 (1.26-1.41) |
| Women | 163,697 (88.2) | 15,791 (69.3) | 0.91 (0.89-0.92) | 64,924 (26.5) | 8,321 (25.2) | 1.09 (1.07-1.12) | 8,799 (9.2) | 1,439 (12.6) | 1.30 (1.23-1.37) |
|  |  |  |  |  |  |  |  |  |  |
| **Race** |  |  |  |  |  |  |  |  |  |
| White | 311,918 (99.7) | 37,180 (83.6) | 0.92 (0.91-0.93) | 108,910 (28.7) | 16,765 (29.1) | 1.10 (1.09-1.12) | 13,964 (9.8) | 2,671 (14.7) | 1.32 (1.27-1.38) |
| Black | 47,969 (105.0) | 5,156 (88.3) | 0.89 (0.86-0.92) | 16,655 (34.2) | 2,308 (34.0) | 1.06 (1.02-1.11) | 1,725 (10.4) | 310 (15.7) | 1.32 (1.16-1.49) |
| Asian | 20,870 (83.4) | 1,813 (71.6) | 0.95 (0.91-1.00) | 9,131 (29.1) | 935 (26.5) | 1.12 (1.04-1.20) | 1,031 (9.4) | 144 (12.1) | 1.26 (1.06-1.51) |
| Other | 2,451 (96.3) | 139 (71.2) | 0.89 (0.75-1.06) | 817 (27.5) | 65 (25.1) | 1.08 (0.84-1.40) | 101 (9.6) | 8 (10.3) | 1.24 (0.58-2.67) |
|  |  |  |  |  |  |  |  |  |  |
| **Cohabitation status** |  |  |  |  |  |  |  |  |  |
| No | 183,071 (112.7) | 19,129 (91.9) | 0.91 (0.90-0.93) | 57,135 (31.6) | 7,996 (31.4) | 1.08 (1.06-1.11) | 6,957 (11.2) | 1,181 (16.1) | 1.29 (1.21-1.37) |
| Yes | 184,780 (88.6) | 23,375 (77.9) | 0.92 (0.91-0.93) | 73,224 (27.7) | 11,288 (28.2) | 1.12 (1.10-1.14) | 9,309 (9.0) | 1,856 (14.0) | 1.35 (1.29-1.42) |
| Unknown | 15,357 (102.1) | 1,784 (80.3) | 0.90 (0.86-0.95) | 5,154 (29.5) | 789 (28.0) | 1.05 (0.97-1.13) | 555 (9.3) | 96 (12.2) | 1.20 (0.96-1.50) |
|  |  |  |  |  |  |  |  |  |  |
| **% of High-school education in county of residence** |  |  |  |  |  |  |  |  |  |
| Lowest tertile | 78,357 (105.3) | 8,216 (87.7) | 0.91 (0.89-0.93) | 25,691 (31.0) | 3,448 (30.6) | 1.10 (1.06-1.14) | 2,783 (10.2) | 444 (14.3) | 1.26 (1.14-1.40) |
| Middle tertile | 140,253 (101.2) | 14,207 (84.2) | 0.91 (0.89-0.92) | 48,707 (30.0) | 6,246 (29.7) | 1.08 (1.05-1.11) | 5,748 (10.2) | 885 (15.2) | 1.30 (1.21-1.40) |
| Highest tertile | 164,598 (95.2) | 21,865 (81.7) | 0.92 (0.91-0.93) | 61,115 (28.1) | 10,379 (28.9) | 1.11 (1.09-1.13) | 8,290 (9.4) | 1,804 (14.4) | 1.35 (1.28-1.42) |
|  |  |  |  |  |  |  |  |  |  |
| **Cost of living adjusted median household income in county of residence** |  |  |  |  |  |  |  |  |  |
| Lowest tertile | 37,218 (108.0) | 3,171 (89.6) | 0.90 (0.87-0.94) | 12,068 (32.8) | 1,381 (35.5) | 1.17 (1.11-1.24) | 1,312 (10.5) | 162 (16.4) | 1.42 (1.20-1.67) |
| Middle tertile | 61,854 (104.1) | 6,558 (85.6) | 0.91 (0.89-0.93) | 20,101 (30.9) | 2,745 (31.2) | 1.10 (1.05-1.14) | 1,957 (11.1) | 270 (15.5) | 1.26 (1.10-1.43) |
| Highest tertile | 284,136 (97.3) | 34,559 (82.6) | 0.92 (0.91-0.93) | 103,344 (28.6) | 15,947 (28.7) | 1.10 (1.08-1.11) | 13,552 (9.6) | 2,701 (14.5) | 1.33 (1.28-1.39) |
|  |  |  |  |  |  |  |  |  |  |
| **Calendar period at diagnosis** |  |  |  |  |  |  |  |  |  |
| 1988-1992 | 38,430 (106.4) | 4,084 (98.4) | 0.95 (0.92-0.98) | 13,811 (30.3) | 1,756 (31.0) | 1.08 (1.03-1.14) | 2,437 (9.4) | 472 (15.2) | 1.38 (1.25-1.53) |
| 1993-1997 | 38,430 (106.4) | 4,084 (98.4) | 0.95 (0.92-0.98) | 13,811 (30.3) | 1,756 (31.0) | 1.08 (1.03-1.14) | 2,437 (9.4) | 472 (15.2) | 1.38 (1.25-1.53) |
| 1998-2002 | 77,247 (105.0) | 6,237 (91.1) | 0.90 (0.88-0.92) | 28,815 (29.9) | 3,068 (31.3) | 1.09 (1.05-1.13) | 5,170 (9.8) | 749 (14.3) | 1.25 (1.16-1.35) |
| 2003-2006 | 82,948 (100.3) | 8,665 (84.0) | 0.91 (0.89-0.93) | 32,522 (28.5) | 4,613 (28.7) | 1.10 (1.06-1.13) | 5,576 (9.8) | 1,109 (13.7) | 1.27 (1.19-1.36) |
| 2007-2010 | 83,923 (94.2) | 11,331 (77.5) | 0.91 (0.89-0.93) | 34,709 (27.2) | 6,438 (27.1) | 1.11 (1.08-1.14) | 1,947 (10.7) | 531 (16.3) | 1.44 (1.30-1.58) |
| 2011-2014 | 38,430 (106.4) | 4,084 (98.4) | 0.95 (0.92-0.98) | 13,811 (30.3) | 1,756 (31.0) | 1.08 (1.03-1.14) | 2,437 (9.4) | 472 (15.2) | 1.38 (1.25-1.53) |
|  |  |  |  |  |  |  |  |  |  |
| **Tumor stage** |  |  |  |  |  |  |  |  |  |
| Localized | 23,747 (25.5) | 4,715 (25.6) | 0.97 (0.94-1.00) | 27,266 (13.9) | 6,565 (18.3) | 1.21 (1.18-1.25) | 7,346 (8.0) | 1,773 (13.4) | 1.42 (1.35-1.50) |
| Regional | 66,720 (56.9) | 8,512 (55.3) | 0.94 (0.92-0.96) | 46,301 (28.5) | 6,522 (31.5) | 1.12 (1.09-1.15) | 6,225 (10.6) | 942 (15.6) | 1.26 (1.18-1.35) |
| Distant | 270,553 (173.2) | 28,473 (171.2) | 0.91 (0.89-0.92) | 53,633 (63.8) | 5,782 (64.4) | 1.00 (0.97-1.02) | 2,340 (15.6) | 265 (19.2) | 1.04 (0.91-1.18) |
| Unknown | 22,188 (115.0) | 2,588 (99.4) | 0.89 (0.85-0.92) | 8,313 (43.0) | 1,204 (44.2) | 1.00 (0.94-1.06) | 910 (14.3) | 153 (20.1) | 1.20 (1.01-1.43) |
|  |  |  |  |  |  |  |  |  |  |
| **Histology** |  |  |  |  |  |  |  |  |  |
| Small cell | 61,664 (121.6) | 6,173 (130.2) | 0.96 (0.93-0.99) | 19,376 (57.8) | 1,789 (60.5) | 1.04 (0.99-1.09) | 1,144 (11.7) | 126 (18.3) | 1.29 (1.07-1.56) |
| Squamous cell | 69,948 (83.9) | 9,529 (73.6) | 0.94 (0.92-0.96) | 30,064 (29.3) | 5,216 (32.3) | 1.15 (1.12-1.19) | 4,148 (11.6) | 811 (17.7) | 1.39 (1.29-1.50) |
| Adenocarcinoma | 106,873 (82.8) | 12,782 (67.9) | 0.92 (0.91-0.94) | 44,976 (26.8) | 6,874 (25.8) | 1.08 (1.05-1.11) | 5,740 (9.9) | 1,177 (14.5) | 1.31 (1.22-1.39) |
| Other | 144,723 (117.9) | 15,804 (95.7) | 0.88 (0.87-0.90) | 41,097 (26.0) | 6,194 (27.5) | 1.11 (1.08-1.14) | 5,789 (8.5) | 1,019 (12.7) | 1.31 (1.23-1.40) |
|  |  |  |  |  |  |  |  |  |  |
| **Tumor size** |  |  |  |  |  |  |  |  |  |
| 0 to 3 cm | 67,847 (52.0) | 9,744 (41.8) | 0.94 (0.92-0.96) | 42,064 (18.8) | 8,501 (21.3) | 1.18 (1.16-1.21) | 8,166 (8.7) | 1,858 (13.6) | 1.38 (1.31-1.45) |
| >3 to 5 cm | 72,100 (83.3) | 8,734 (77.9) | 0.97 (0.94-0.99) | 32,258 (30.7) | 4,488 (33.2) | 1.11 (1.07-1.14) | 3,883 (10.5) | 616 (15.3) | 1.24 (1.14-1.35) |
| >5 to 7 cm | 46,671 (108.8) | 5,234 (112.7) | 0.96 (0.93-0.99) | 15,196 (37.4) | 1,705 (40.6) | 1.05 (0.99-1.10) | 1,340 (10.0) | 160 (14.1) | 1.21 (1.02-1.43) |
| >7 cm | 43,213 (136.3) | 4,137 (138.5) | 0.93 (0.90-0.96) | 10,719 (43.6) | 1,020 (45.9) | 1.07 (1.00-1.14) | 754 (10.4) | 100 (17.0) | 1.37 (1.11-1.70) |
| Unknown | 153,377 (162.8) | 16,439 (151.2) | 0.90 (0.88-0.91) | 35,276 (51.7) | 4,359 (52.3) | 1.02 (0.99-1.05) | 2,678 (13.5) | 399 (19.5) | 1.24 (1.12-1.38) |
|  |  |  |  |  |  |  |  |  |  |
| **Tumor grade** |  |  |  |  |  |  |  |  |  |
| Well differentiated | 6,445 (30.2) | 1,056 (26.4) | 0.95 (0.89-1.02) | 5,156 (11.7) | 1,164 (14.2) | 1.27 (1.19-1.35) | 1,386 (7.1) | 343 (11.7) | 1.40 (1.24-1.58) |
| Moderately differentiated | 30,881 (47.2) | 4,437 (40.2) | 0.96 (0.93-0.99) | 21,509 (19.0) | 4,032 (21.0) | 1.17 (1.13-1.21) | 4,274 (9.5) | 865 (13.4) | 1.26 (1.17-1.36) |
| Poorly differentiated | 91,644 (89.9) | 10,511 (76.8) | 0.93 (0.91-0.95) | 36,056 (28.0) | 5,439 (29.8) | 1.13 (1.10-1.16) | 4,943 (10.0) | 931 (16.0) | 1.38 (1.29-1.49) |
| Undifferentiated | 29,719 (113.2) | 2,774 (113.9) | 0.97 (0.93-1.01) | 10,077 (41.8) | 963 (41.6) | 1.10 (1.03-1.17) | 1,015 (10.1) | 145 (16.6) | 1.48 (1.24-1.77) |
| Unknown | 224,519 (131.3) | 25,510 (116.7) | 0.90 (0.88-0.91) | 62,715 (41.1) | 8,475 (41.7) | 1.03 (1.01-1.06) | 5,203 (10.8) | 849 (16.0) | 1.27 (1.18-1.36) |
|  |  |  |  |  |  |  |  |  |  |
| **Surgery**^*^ |  |  |  |  |  |  |  |  |  |
| No | 295,380 (133.4) | 34,169 (119.9) | 0.90 (0.89-0.91) | 84,299 (52.5) | 11,305 (52.1) | 1.01 (0.99-1.03) | 5,111 (17.3) | 739 (23.1) | 1.10 (1.02-1.19) |
| Yes | 17,077 (17.3) | 3,093 (17.7) | 1.03 (0.99-1.07) | 25,941 (11.7) | 5,704 (15.3) | 1.31 (1.28-1.35) | 7,492 (7.7) | 1,641 (12.3) | 1.39 (1.31-1.46) |
| Unknown | 4,282 (140.9) | 316 (130.6) | 0.88 (0.78-0.99) | 1,144 (46.3) | 89 (42.1) | 0.92 (0.74-1.15) | 90 (11.4) | 9 (21.4) | 1.64 (0.77-3.49) |
|  |  |  |  |  |  |  |  |  |  |
| **Radiation therapy** |  |  |  |  |  |  |  |  |  |
| No/Unknown | 222,544 (104.8) | 27,546 (83.4) | 0.91 (0.90-0.92) | 66,685 (21.7) | 11,807 (23.4) | 1.13 (1.11-1.15) | 11,151 (8.5) | 2,412 (13.4) | 1.36 (1.30-1.42) |
| Yes | 160,664 (92.6) | 16,742 (83.7) | 0.94 (0.93-0.96) | 68,828 (44.4) | 8,266 (46.2) | 1.05 (1.03-1.08) | 5,670 (14.1) | 721 (21.4) | 1.21 (1.12-1.31) |
|  |  |  |  |  |  |  |  |  |  |
| **Chemotherapy** |  |  |  |  |  |  |  |  |  |
| No/Unknown | 246,638 (121.8) | 30,256 (91.8) | 0.90 (0.89-0.92) | 61,872 (20.9) | 11,741 (23.6) | 1.13 (1.11-1.16) | 11,534 (8.9) | 2,449 (14.1) | 1.36 (1.30-1.42) |
| Yes | 136,570 (74.4) | 14,032 (69.9) | 0.96 (0.94-0.98) | 73,641 (44.1) | 8,332 (44.9) | 1.06 (1.03-1.08) | 5,287 (12.4) | 684 (17.1) | 1.21 (1.11-1.31) |

N, number of deaths; IR, incidence rate per 100 person-years; HR, hazard ratio; CI, confidence interval; LCa, Lung cancer. HRs were adjusted for age and calendar period at diagnosis, sex, race, cohabitation status, % of high-school education and cost of living in county of residence, tumor stage, histology, tumor grade, surgery, radiation therapy, and chemotherapy, if applicable.

^*^ Information on surgery was available from 1998 onward.
